# Efficacy of Motivational Interviewing to Improve Utilization of Mental Health Services Among Youths With Chronic Medical Conditions: A Cluster Randomized Clinical Trial

**DOI:** 10.1001/jamanetworkopen.2021.27622

**Published:** 2021-10-01

**Authors:** Christina Reinauer, Anna Lena Platzbecker, Rabea Viermann, Matthias Domhardt, Harald Baumeister, Katharina Foertsch, Hannah Linderskamp, Lisa Krassuski, Doris Staab, Kirsten Minden, Reinhold Kilian, Reinhard W. Holl, Petra Warschburger, Thomas Meißner

**Affiliations:** 1 Department of General Pediatrics, Neonatology and Pediatric Cardiology, University Children's Hospital, Heinrich-Heine-University, Düsseldorf, Germany; 2 Department of Clinical Psychology and Psychotherapy, Ulm University, Ulm, Germany; 3 Department of Pediatric Pneumology and Immunology, University Children's Hospital Charité of Humboldt University, Berlin, Germany; 4 German Rheumatism Research Centre Berlin, and Charité, University Medicine, Berlin, Germany; 5 Department of Psychiatry and Psychotherapy II, University of Ulm and BKH Günzburg, Günzburg, Germany; 6 Institute for Epidemiology and Medical Biometry, ZIBMT, University of Ulm, Ulm, Germany; 7 Department of Psychology, Counseling Psychology, University of Potsdam, Potsdam, Germany

## Abstract

**Importance:**

Despite the high prevalence of anxiety and depression in youths with chronic medical conditions (CMCs), physicians encounter substantial barriers in motivating these patients to access mental health care services.

**Objective:**

To determine the efficacy of motivational interviewing (MI) training for pediatricians in increasing youths’ use of mental health care.

**Design, Setting, and Participants:**

The COACH-MI (Chronic Conditions in Adolescents: Implementation and Evaluation of Patient-Centered Collaborative Healthcare—Motivational Interviewing) study was a single-center cluster randomized clinical trial at the University Children’s Hospital specialized outpatient clinics in Düsseldorf, Germany. Treating pediatricians were cluster randomized to a 2-day MI workshop or treatment as usual (TAU). Patient recruitment and MI conversations occurred between April 2018 and May 2020 with 6-month follow-up and 1-year rescreening. Participants were youths aged 12 to 20 years with CMCs and comorbid symptoms of anxiety and depression; they were advised by their MI-trained or untrained physicians to access psychological counseling services. Statistical analysis was performed from October 2020 to April 2021.

**Interventions:**

MI physicians were trained through a 2-day, certified MI training course; they recommended use of mental health care services during routine clinical appointments.

**Main Outcomes and Measures:**

The primary outcome of uptake of mental health care services within the 6-month follow-up was analyzed using a logistic mixed model, adjusted for the data’s cluster structure. Uptake of mental health services was defined as making at least 1 appointment by the 6-month follow-up.

**Results:**

Among 164 youths with CMCs and conspicuous anxiety or depression screening, 97 (59%) were female, 94 (57%) had MI, and 70 (43%) had TAU; the mean (SD) age was 15.2 (1.9) years. Compared with patients receiving TAU, the difference in mental health care use at 6 months among patients whose physicians had undergone MI training was not statistically significant (odds ratio [OR], 1.96; 95% CI, 0.98-3.92; *P* = .06). The effect was moderated by the subjective burden of disease (*F*_2,158_ = 3.42; *P* = .04). Counseling with an MI-trained physician also led to lower anxiety symptom scores at 1-year rescreening (*F*_1,130_ = 4.11; *P* = .045). MI training was associated with longer conversations between patients and physicians (30.3 [16.7] minutes vs 16.8 [12.5] minutes; *P* < .001), and conversation length significantly influenced uptake rates across conditions (OR, 1.03; 95% CI, 1.01-1.06; *P* = .005).

**Conclusions and Relevance:**

In this study, use of MI in specialized pediatric consultations did not increase the use of mental health care services among youths with CMCs but did lead to longer patient-physician conversations and lower anxiety scores at 1 year. Additional research is required to determine whether varying scope and duration of MI training for physicians could encourage youths with CMCs to seek counseling and thus improve integrated care models.

**Trial Registration:**

German Trials Registry: DRKS00014043

## Key Points

**Question:**

Does pediatrician training in motivational interviewing (MI) facilitate the use of mental health care services for youths with chronic medical conditions?

**Findings:**

In this cluster randomized trial with 164 youths with chronic medical conditions and comorbid anxiety or depression, training physicians in MI did not significantly improve uptake rates of psychological counseling among youths compared with usual care. MI training was associated with longer patient-physician conversations and lower anxiety scores at 1-year rescreening.

**Meaning:**

More research is needed to determine whether training pediatricians in MI and allotting more consultation time to discuss mental health could facilitate use of counseling services by youths with chronic medical conditions.

## Teaser

This cluster randomized clinical trial examined the efficacy of pediatricians using motivational interviewing (MI) vs treatment as usual in increasing youths’ utilization of mental health care.

## Introduction

Youths with chronic medical conditions (CMCs) represent a high-risk group for comorbid anxiety, depression, or behavioral problems, with a reported prevalence of 10% to 42%.^1,2,3,4,5,6^ Concomitant psychological concerns compromise therapy for somatic disorders, health-related behavior, medication adherence, and increase long-term health risks into adulthood.^7,8,9,10,11^ Early detection of mental health problems and timely initiation of evidence-based treatment is important.^12,13,14,15,16,17^ However, physicians can encounter substantial individual barriers, and referring youths who lack intrinsic motivation to psychological counseling typically fails. Youths tend to underutilize services, fearing stigmatization and preferring autonomy,^18,19,20,21^ and only a fraction receives adequate treatment.^22,23,24,25^ Growing evidence exists for the effectiveness of integrated mental health care for pediatric in- and outpatient care. Identification of psychological disorders and referral to adequate treatment options are important factors for therapy uptake.^26^ Validated tools to detect comorbid mental health problems and brief psychological interventions, such as motivational interviewing (MI) are applicable.^27^ MI is an evidence-based, collaborative counseling technique, exploring intrinsic motivation and ambivalence.^28,29^ It has been shown to facilitate uptake of therapy in suicidal patients^30^ and the uptake of cognitive behavioral therapy or Internet-based interventions for depression in adolescents.^31,32,33,34^

Published data suggest that MI can be implemented into clinical routines to improve young patients’ mental health care services utilization.^35,36^ Our study aimed to determine the efficacy of MI training for pediatricians in increasing adolescents’ use of mental health care services.

## Methods

The COACH-MI study was conducted within the research framework of the COACH consortium (Chronic Conditions in Adolescents: Implementation and Evaluation of Patient-Centered Collaborative Healthcare), which aimed to increase awareness of and access to mental health care for youths aged 12 to 20 years with CMCs. The study protocol was published previously.^37^ The study received ethical approval from the University of Düsseldorf institutional review board.

### Design, Setting, and Participants

This single-center cluster randomized clinical trial was conducted at the University Children’s Hospital in Düsseldorf, Germany. Patient recruitment occurred between April 2018 and May 2020 with a 6-month follow-up and 1-year rescreening.

Clusters were constituted by physicians in specialized outpatient clinics who were randomized into either MI or treatment as usual (TAU) groups. Participating physicians (n = 51 randomized; n = 37 saw patients in the study setting; n = 18 [49%] MI; n = 19 [51%] TAU; n = 22 [60%] female, n = 19 [51%] senior physicians or fellows) had no previous training in psychiatry, psychotherapy, or MI, and they provided written informed consent. There were no exclusion criteria for physicians.

Youths aged 12 to 20 years with CMCs (condition duration more than 1 year and requiring continuous medical treatment) were invited to complete questionnaires on depression, anxiety, and medication adherence before their routine appointment in the specialized pediatric outpatient clinics (pulmonology, diabetes and endocrinology, metabolic diseases, cardiology, gastroenterology, rheumatology and immunology, and neurology). The study included 164 patients with conspicuous screening results. Written informed consent was obtained from patients and legal guardians (for patients aged younger than 18 years). Patient exclusion criteria included regular psychotherapy, psychosis, acute suicidal ideations, diagnosis of substance abuse, severe intellectual disability (IQ < 70), and language limitations. No patient was on psychotropic medication.

### Assessments

Anxiety and depression symptoms were assessed using the Generalized Anxiety Disorder 7-item scale (GAD-7)^38^ and the Patient Health Questionnaire 9 (PHQ-9).^39^ The GAD-7 includes 7 prevalent symptoms of generalized anxiety disorder, while the PHQ-9 includes 9 criteria for depression, including 1 item about ideation of suicidal behavior or self-harm (item 9). Both questionnaires refer to the 2 weeks prior to assessment, and items score from 0 (not at all) to 3 (nearly every day)*.* A positive screening result was defined as a score greater than or equal to 7 in either GAD-7 or PHQ-9 or a positive answer to item 9 of the PHQ-9 (ie, thoughts that you would be better off dead, or thoughts of hurting yourself in some way at least on several days [score ≥1]). A red flag, indicating severe symptoms, was displayed to physicians for patients who scored at least 15 of 21 points on the GAD-7, at least 20 of 27 points on the PHQ-9, or who gave a positive response to item 9. Consultations evaluated suicidality using the Columbia-Suicide Severity Rating Scale.^40^ Medication adherence was evaluated using the German version of the Medication Adherence Report Scale (MARS-D) questionnaire (maximum score 25).^41^ Medical records provided anthropometric data included age, sex, height, weight, body mass index (BMI; calculated by weight in kilograms divided by height in meters squared), and disease-related parameters. Patients reported subjective symptom burden, pain occurrence, and everyday-life restrictions (all Likert scale, 1 = not at all to 4 = nearly every day or extremely stressful), missed school or workdays within the last 6 months, and daily treatment duration. Data on race and ethnicity were not collected. The primary outcome was evaluated 6 months postintervention during a standardized follow-up phone call. Participants and/or their legal guardians were interviewed about mental health care service use. Rescreening with the same questionnaires was scheduled approximately 1 year postintervention.

### Randomization

Pediatricians (n = 51) were cluster-randomized into the MI intervention (n = 25) or TAU (n = 26) groups by an independent biostatistician. Outpatient clinic specialization and expected cluster size (small or big) were considered using the minimization (dynamic allocation) method^42^ to neutralize imbalances between groups (Supplement 1). Patient allocation depended on the treating physician’s randomization into MI or TAU group; 94 patients saw an MI-trained pediatrician, and 70 received TAU consultation.

### Intervention

We established MI training to enable physicians to deliver an MI-based ultrashort intervention aiming at patients’ motivation toward claiming mental health care. Pediatricians randomized into the MI training group received a 2-day training course conducted by a Motivational Interviewing Network of Trainers (MINT) certified trainer (Supplement 1). They signed a confidentiality agreement to prevent potential trial group contamination. One-day MI booster sessions were conducted 1 year after study initiation. All pediatricians engaged in counseling conversations with their patients on conspicuous screening results and recommended the use of mental health services. MI physicians offered a voluntary second visit to further discuss the topic with their patients, whereas TAU physicians were not advised about second appointments. Patients were blinded regarding a physician’s training, and all received the same standardized written feedback on assessments and contact information for mental health care appointments. If mutual consent was given, conversations were audio recorded to analyze treatment fidelity. Physicians self-reported conversation length and semiquantitative use of MI-consistent techniques after each consultation (6 basic MI techniques: open-ended questions, reflective listening, affirmations, advice with permission, creating collaboration, and emphasizing autonomy and control were graded from 0 = not used to 2 = often used*,* for a maximum score of 12 points).

### Outcomes

The primary outcome measured was the use of mental health care services, defined as making at least 1 appointment (vs no appointment) by the 6-month follow-up. Patients on waiting lists were counted as positive outcomes. Secondary outcomes included baseline changes in GAD-7, PHQ-9, and MARS-D scores; the number of psychological sessions attended within the follow-up interval; sex-specific analyses; and severe adverse events (SAEs) as well as missed clinical visits evaluation.

Exploratory analysis included the influence of clinical parameters and conversation length on the primary outcome; reasons for not seeking psychological counseling; the physicians’ MI-consistency; satisfaction with the intervention and use of second MI/TAU appointments; and the type of mental health service utilized.

### Statistical Analysis

The rate of mental health care uptake for the TAU group was estimated at 10%,^43^ and an increase to 30% for the MI group was expected. A sample size of n = 62 per trial group was calculated with NQuery Advanced version 8.1 (Statistical Solutions) using a 2-sided χ^2^ test with power at 80% and significance level at 5%. A cluster size of 5 patients per physician, estimated intracluster correlation coefficient of 2.5%, corrected by 10% for cluster effects, resulted in a sample size of n = 69 per group. Adjusting for 15% dropout, we aimed for n = 162 (n = 81 per group) patients to participate in the study (Supplement 1).^37^ Statistical analyses were performed with SPSS Statistics version 26 (IBM) from October 2020 to April 2021. MI efficacy was examined in an intention-to-treat approach. For imputation, a logistic regression model was used for the primary outcome models (including the variables sex, age, intervention, PHQ-9 and GAD-7 score, pain, burden of disease, and conversation length). The primary outcome was analyzed using a logistic mixed model, adjusting for the data’s cluster structure,^44^ age, and sex. Secondary and exploratory analyses used logistic mixed models, nonparametric tests, and mixed analysis of variance. For comparison of SAE rates, Fisher exact test was used. Means and SDs were reported for symmetrical data, whereas medians and IQRs were used for nonsymmetrical data. Two-sided *P* < .05 indicated statistical significance. We obtained only a few audio recordings, insufficient for a structured evaluation.

## Results

**Figure 1.**
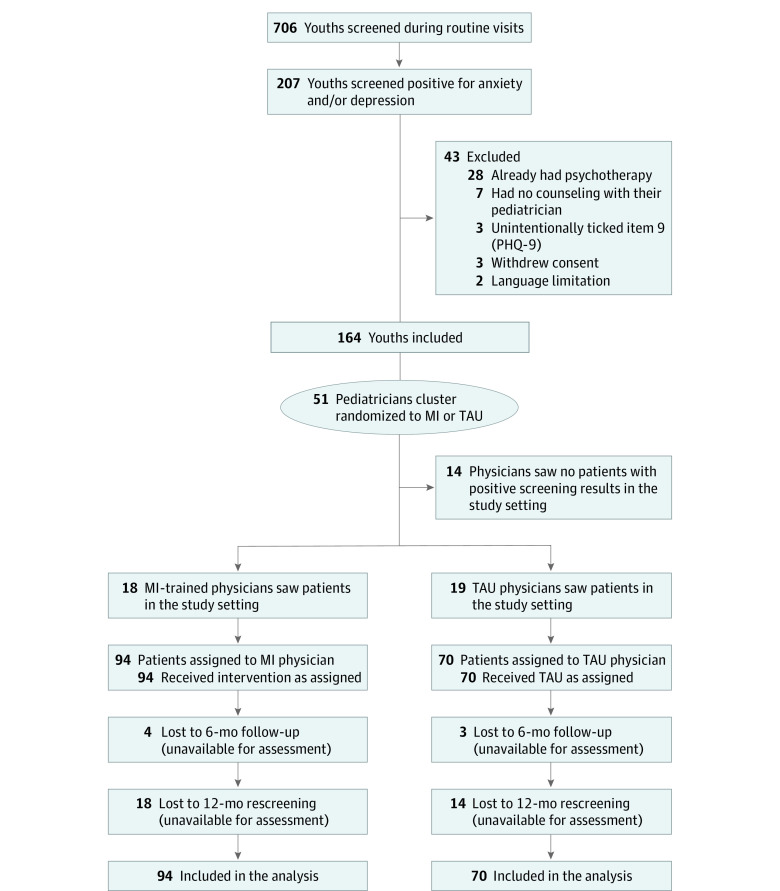
CONSORT Flowchart of the Study The patients’ allocations were based on their treating pediatricians’ randomization to MI or TAU groups. At follow-up 7 patients (4%) and at rescreening 32 patients (19.5%) were lost to follow-up. MI indicates motivational interviewing; PHQ-9, Patient Health Questionnaire 9; TAU, treatment as usual.

Of 706 youths with CMCs, the study included 164 patients (97 [59%] were female youths, 94 [57%] had MI, and 70 [43%] had TAU); the mean (SD) age was 15.2 [1.9] years. Among these 164 patients, 62 had positive screening results for depression, 25 screened positive for anxiety symptoms, and 68 screened positive for both depression and anxiety, and 9 had a positive item 9 on the PHQ-9 (Figure 1). Patient variables for the MI and TAU pediatrician groups were equivalent at baseline (Table 1). Most patients had mild to moderately conspicuous mean (SD) screening results (GAD-7, 7.4 [0.3]; PHQ-9, 8.8 [0.3]). A total of 71 patients (MI: n = 43; TAU: n = 28) scored at least 10 points on either of the screening tests, indicating moderately severe depression or anxiety. Seven patients had red flags from screening scores. Altogether, 55 patients had a positive item 9.

**Table 1.** Patient Characteristics of MI and TAU Counseling Groups

Variable	Mean (SD)		
	All (n = 164)	MI (n = 94)	TAU (n = 70)
Age	15.2 (1.9)	15.2 (2.0)	15.2 (1.9)
Sex, female, No. (%)	97 (59)	51 (54)	46 (65)
BMI^a^	0.3 (1.1)	0.2 (1.1)	0.3 (1.1)
Hemoglobin A_1c_, % (n = 42)	8.0 (1.5)	8.2 (1.7)	7.7 (1.3)
GAD-7 score	7.4 (3.6)	7.7 (3.6)	6.9 (3.5)
PHQ-9 score	8.8 (3.6)	8.7 (3.7)	8.9 (3.5)
MARS-D score	22.0 (3.1)	21.6 (3.4)	22.5 (2.6)
Subjective burden of disease	2.2 (0.8)	2.2 (0.9)	2.1 (0.7)
Pain^b^	2.1 (0.9)	2.2 (0.8)	2.0 (0.9)
Everyday life restrictions^b^	1.8 (0.9)	1.8 (0.9)	1.6 (0.9)
Absent school or workdays, median (IQR)^c^	10.0 (3.3-24.3)	12.5 (4-25.5)	7.0 (3-17)
Time requirement for therapy, median (IQR)^d^	5.0 (1-15)	5.0 (2-20)	5.0 (5-15)

Abbreviations: BMI, body mass index; GAD-7, Generalized Anxiety Disorder 7-item scale; MI, motivational interviewing; PHQ-9, Patient Health Questionnaire 9; TAU, treatment as usual.

^a^ BMI is calculated at weight in kilograms divided by height in meters squared.

^b^ Pain occurrence and everyday life restrictions (Likert scale, 1 = not at all to 4 = nearly every day or extremely stressful).

^c^ Absent school or workdays during last 6 months.

^d^ Time requirement for therapy related to chronic medical condition (minutes per day).

### Primary Outcome

We found a difference between the 2 groups concerning mental health care access at follow-up but it did not meet the threshold for statistical significance (MI: 36 patients [40.0%] vs TAU: 18 patients [26.8%]; adjusted odds ratio [OR], 1.96 [95% CI, 0.98-3.92]; *P* = .06) (Table 2). Among positive outcomes, 6 patients were on waiting lists, and 4 had postponed appointments. For patients who scored at least 10 on either test, the adjusted OR for treatment initiation in the MI group was 2.08 (95% CI, 0.74-5.83; *P* = .16).

**Table 2.** Uptake of Mental Health Services After Conversations With MI or TAU Physicians at 6-Month Follow-up

Characteristic	No. (%)		
	MI (n = 94)	TAU (n = 70)	Total (n = 164)
Uptake of mental health services	36 (40.0)	18 (26.8)	54 (34.4)
No uptake	54 (60.0)	49 (73.2)	103 (65.6)
Lost to follow-up, No.	4	3	7

Abbreviations: MI, motivational interviewing; TAU, treatment as usual.

### Secondary Outcomes

**Figure 2.**
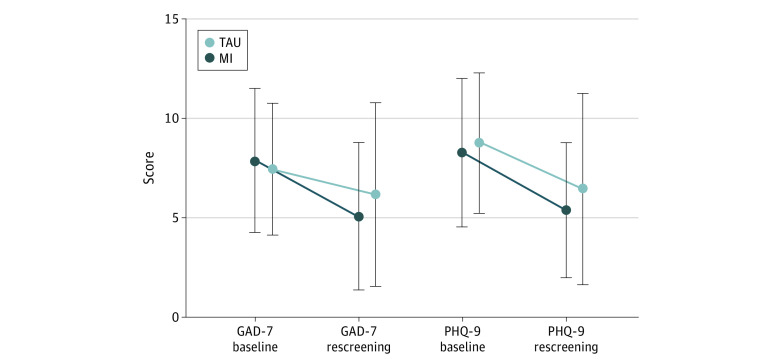
GAD-7 and PHQ-9 Scores at Baseline and Rescreening for MI and TAU Patients Anxiety and depression significantly improved upon rescreening in both conditions for MI (GAD-7, 7.9 [3.6] vs 5.1 [3.7]; *P* < .001; PHQ-9, 8.3 [3.7] vs 5.4 [3.4]; *P* < .001) and TAU (GAD-7, 7.5 [3.3] vs 6.2 [4.6]; *P* = .036; PHQ-9, 8.8 [3.5] vs 6.5 [4.8]; *P* = .002). GAD-7 improved better in the MI group (time point [baseline vs rescreening] × intervention effect), compared with TAU (*P* = .045). GAD-7 indicates Generalized Anxiety Disorder 7-item scale; MI, motivational interviewing; PHQ-9, Patient Health Questionnaire 9; TAU, treatment as usual.

Anxiety and depression scores significantly improved upon rescreening in both groups independent of the intervention, leading to a high remission rate of 53.6% (70 of 132) (MI: 50% [38 of 76]; TAU: 57.1% [32 of 56]). Patients’ anxiety symptom scores further improved after physicians’ MI training (time point [baseline vs rescreening] × intervention effect; *F*_1,130_ = 4.11; *P* = .045), whereas depression symptoms did not (*F*_1,130_ = 0.54; *P* = .46; Figure 2). Mean (SD) adherence scores (MARS-D) were stable before and after intervention in both conditions (22.2 [2.7] vs 22.5 [2.7]; *F*_1,130_ = 1.75; *P* = .19; time point × intervention effect: *F*_1,129_ = 0.25; *P* = .62).

The total number of psychological counseling appointments completed at 6 months did not significantly differ between the MI and TAU groups; 6 MI patients (18%) and 5 TAU patients (28%) had at least one appointment; 12 MI patients (33%) and 5 TAU patients (28%) had 2 to 9 appointments; and 9 MI patients (25%) and 6 TAU patients (33%) had more than 10 appointments (*U* = 210; *z* = −0.499; *P* = .62). Reasons for not seeking psychological support are listed in Table 3. Missed clinical visits during follow-up were similar in both groups.

**Table 3.** Reasons for Not Seeking Psychological Support at Follow-up

Reasons for not making an appointment (multiple options possible)	No.
Total No.	103
No perceived need for further psychological support	77
A critical attitude toward psychologists (only for the mentally ill)	3
Did not have time to make an appointment	12
Forgot to make an appointment	5
Insecurity about mental health care (we did not know what to expect)	3
Psychological support will have no benefit	4
My child didn't want me to make an appointment (parents' reports, n = 58)	11
Other	5

Female patients did not have significantly higher mean (SD) screening scores (PHQ-9: 9.1 [3.8] vs 8.4 [3.3]; *P* = .21; GAD-7: 7.6 [3.5] vs 7.0 [3.7]; *P* = .25), reported symptom burden (2.3 [0.8] vs 2.0 [0.7]; *P* = .07), or rates of suicidal ideation (item 9: 35 female patients [36.1%] vs 20 male patients [29.9%]; *P* = .41), nor were female patients in general significantly more likely to make an appointment (OR, 1.19 [95% CI, 0.61-2.35]; *P* = .61).

SAEs were monitored throughout the study. Patients had 35 hospital admissions, primarily related to CMCs, and unrelated to the intervention. Four related SAEs were reported, regarding an inpatient crisis intervention within a pediatric or psychiatric department, with no significant differences between the treatment groups (MI, 3 patients; TAU, 1 patient; *P* = .64, Fisher exact test).

### Exploratory Analysis

Individual effects on the outcome, uptake of mental health treatment, were observed for PHQ-9 scores (OR, 1.11; 95% CI, 1.01-1.21; *P* = .04) or GAD-7 scores (OR, 1.13; 95% CI, 1.02-1.24; *P* = .02), patient-reported burden of disease (OR, 1.64; 95% CI, 1.06-2.51; *P* = .03) and pain occurrence (OR, 1.59; 95% CI, 1.07-2.36; *P* = .02). Adding these covariates to the primary outcome analysis, the MI effect was not significant (OR, 1.82; 95% CI, 0.88-3.76; *P* = .10). In multivariate analysis the effect of MI was not moderated by most clinical characteristics (anxiety and depression symptoms, sex, age, standardized BMI, disease category, MARS-D, pain occurrence, daily treatment duration and everyday life restrictions), but only by the subjective burden of disease (intervention × burden of disease; *F*_2,158_ = 3.42; *P* = .04).

MI conversations had significantly longer mean (SD) times than TAU conversations (30.3 [16.7] minutes vs 16.8 [12.5] minutes; *P* < .001). As an individual parameter, conversation length was significantly associated with uptake rates (OR, 1.03; 95% CI, 1.01-1.06; *P* = .005). The influence of conversation length on the primary outcome model was more pronounced in the MI group (OR, 1.03; 95% CI, 1.00-1.06; *P* = .052) compared with TAU (OR, 1.03; 95% CI, 0.98-1.07; *P* = .25). Including an interaction term between conversation length and intervention (MI vs TAU) into our model showed no significant modifying effect of conversation length on the effect of MI on uptake.

When rating the statement “Conversation was helpful” on a Likert scale (0 = not at all to 4 = very helpful), satisfactory mean (SD) scores for both MI and TAU sessions were reported by patients (MI: 3.25 [0.99] vs TAU: 2.98 [1.19]; *P* = .17), parents (MI: 3.32 [0.96] vs TAU: 3.03 [1.05]; *P* = .21), and physicians (MI: 2.82 [0.91] vs TAU: 2.72 [0.82]; *P* = .55). MI physicians’ mean (SD) self-assessment scores were 9.8 (1.3) of 12 points for the use of MI-consistent conversation strategies. The optional second appointment with MI pediatricians was attended by 24 (25.5%) patients; no TAU patients attended an extra appointment. Most patients who attempted to obtain mental health care access were successful (81.5% [44 of 54]). First consultations were with a child and adolescent psychotherapist (n = 26), a clinical psychologist (n = 8), a child and adolescent psychiatrist (n = 8), or other psychosocial services (n = 2). Patients did not utilize online counseling, such as Internet-based cognitive behavioral therapy.

## Discussion

This study incorporated MI training and annual anxiety and depression screening into specialized pediatric care for adolescents with CMCs in a collaborative care setting. The focus on mental health care led to higher-than-expected uptake rates, although the results were not statistically significant. Following an MI or TAU counseling session, 40.0% and 26.8% of adolescent patients with CMCs and anxiety or depression symptoms made appointments for psychological counseling, respectively. Discussions of anxiety and depression screening results between pediatricians and patients in outpatient care is novel. Conversation length (including TAU) was longer than expected, suggesting a relevant need to address and discuss mental health topics during routine care. Conversation length and the level of burden of disease had a relevant influence on mental health service-seeking behavior.

We deliberately selected a low cutoff of at least 7 points on the anxiety and depression screening questionnaires for high sensitivity and low-threshold referral to avoid chronic mental health impairments. This approach allowed us to avoid selecting a cohort with exclusively severe symptoms and to also include patients with fewer symptoms. Including mild anxiety or depression might lead to an underestimation of the benefits of MI training. Many patients reported no subjective need for psychological support, reflecting the issues of internal barriers, shame, and desired autonomy. Concurrently, some patients with mild symptoms might not yet require professional support. In the integrated care approach, the treating physicians can already provide valuable advice with appropriate training (eg, MI) for mild psychosocial, emotional, and behavioral disorders. To improve access rates, we recommend maintaining a low cutoff for offering mental health support for at-risk adolescents experiencing mild or subclinical depression and anxiety.

Our cohort showed high remission rates. This result is potentially due to mild cases, a simple intervention effect focusing on mental health, or spontaneous fluctuations over time. Depressive episodes in adolescents often remit spontaneously within a year.^45,46^ Whereas we observed significantly better improvements only in anxiety scores with the MI approach, other research found that physician MI interventions also alleviated depression symptoms.^32,34^ Short MI seminars have been successful in teaching physicians basic MI skills,^36,47,48,49^ but proficiency and long-term experience are associated with its success. MI delivered by a clinical psychologist with postgraduate MI-training improved treatment initiation and participation,^31^ but MI delivered by social workers was only partly effective in the recent STAT-ED intervention for adolescents with suicidal ideations.^50^ Further research is necessary to evaluate the optimal method and duration of successful physician MI training,^51^ such as implementation early in medical school, as well as the effectiveness of different dosages of MI for adolescents in the framework of clinical visits. Research has shown that single sessions or two 15-minute MI sessions enhance motivation for mental health treatment.^52,53^ MI can be implemented in a busy schedule as an add-on conversation technique (additional 15–20 minutes) during regular appointments, and can potentially improve treatment attendance^31,54,55^ and outcomes,^56^ encourage medication adherence,^57,58^ facilitate the transition to adult care,^59^ and reduce risk-taking behaviors.^60^ Researchers have combined MI with limited case management in a collaborative care setting with varying success (eg, incorporating follow-up phone calls by social workers).^36,50^ However, this approach requires additional resources and time from both caretakers and adolescents, a challenge reflected in the low rate of opting for a second appointment (25.5%).

### Future Directions

Although routine screening has been established for certain pediatric diseases,^4,61^ we implemented anxiety and depression screening for all youths with CMCs and offered counseling and advice. Routinely discussing mental health in pediatric care targets prevention instead of intervention and is a cornerstone in enhancing risk perception and reducing prejudice. A resource-strengthening approach without a focus on psychopathology might be beneficial for patients with borderline screening results. No patient participated in e-Health services because they are not yet available in standard care. The rapidly growing availability of evidence-based and Internet-based interventions, as well as health apps, may increase mental health care accessibility for the young digital generation^33,62,63,64^ and reduce the risk of chronification due to prolonged waiting periods, but motivating adolescents to participate remains a challenge.^65^ This topic will be analyzed in the future research of the COACH consortium.^66,67^

### Limitations

Although this study fulfilled high methodological standards, it has some limitations. For ethical reasons, a nonactive control group was not included. Physicians could not be blinded to the intervention condition, because blinding is impossible in trials on psychological interventions.^68^ A major limitation of our study is that comparing MI with TAU is only possible by providing additional time for MI conversations. Thus, the effects of MI and conversation length cannot be clearly separated in the evaluation. Although statistical significance was not obtained, with larger samples the effect of MI may become significant and may be of clinical relevance.

The power analysis included the estimated patient numbers per pediatrician (high or low). We anticipated equal sizes for MI and TAU groups, but differences occurred randomly because patient allocation was based on their pediatricians’ randomization. To avoid this issue, patients would have needed to change physicians after screening and would no longer have seen their regular pediatricians for counseling. Thus, group size difference was not due to systematic error, and we considered this factor unlikely to have distorted the outcomes. Additionally, we could not perform a structured fidelity assessment of audio recordings^69^ because of limited consent from patients and legal guardians for both MI and TAU consultations. We cannot rule out that TAU pediatricians intuitively used some patient-centered conversation techniques. As a proxy to coding for MI fidelity, we used physicians’ self-reports on the use of 6 core MI techniques after each MI conversation. Finally, previous studies have shown that 2-day training courses were sufficient to improve use of MI methods compared with TAU^29,47,48,49,70^ and we aimed at a realistic approach.

## Conclusions

MI training of physicians and its application in the clinical routine of specialized pediatric consultations was not associated with increased access to mental health care services by adolescents with CMCs, but more research is needed. The MI approach may help break down barriers and stigma associated with psychological concerns. Involving pediatricians as facilitators of mental health treatment in a collaborative care setting is a potentially effective and sustainable approach to fill the current treatment gaps.

## 

## Notes

Supplement 1.
Trial Protocol

Supplement 2.
Data Sharing Statement
