# Supplementary material for: Efficacy of Motivational Interviewing to Improve Utilization of Mental Health Services Among Youths With Chronic Medical Conditions: A Cluster Randomized Clinical Trial
Source: JAMA Netw Open. 2021 Oct 1;4(10):e2127622. doi: 10.1001/jamanetworkopen.2021.27622 (PMC8486984; doi:10.1001/jamanetworkopen.2021.27622)
Supplement: Supplement 1. — Trial Protocol [file jamanetwopen-e2127622-s001.pdf]

Supplement 1 - Study Protocol: Efficacy of motivational interviewing to improve utilization of mental health services among adolescents with chronic medical conditions: A cluster-randomized clinical trial

Previous title:
“COACH-MI – Clinical Study on Motivational Interviewing (MI) as a tool to enhance access to mental health treatment in adolescents with chronic medical conditions and need for psychological support”

German title: „COACH-MI – Patientenzentrierte interdisziplinäre Gesundheitsversorgung bei chronisch kranken Jugendlichen – Klinische Studie zur motivierenden Gesprächsführung“

Acronym: COACH-MI

Summary:
This study will be performed along with the implementation of screening for anxiety and depression for all adolescents (age 12-20 years) with chronic diseases treated in the outpatient clinic of the University Children’s Hospital Düsseldorf. A resulting challenge will be to refer adolescents with signs of anxiety and depression to mental healthcare. Within this setting, we designed a cluster randomized controlled trial to determine the efficacy of taching Motivational Interviewing (MI) technique to the treating physicians, and to arrange time within the appointment framework for MI with adolescents with a conspicuous screening. The aim is to improve their utilization of mental healthcare. The focus of this study is on adolescents with chronic medical conditions presenting with symptoms of depression and anxiety but also adherence problems as measured by screening questionnaires. MI is a collaborative, evidence-based counseling technique designed to elicit intrinsic motivation and strengthen the commitment to improve a range of health behaviors. In this monocentric approach in a center with seven specialized outpatient clinics, n=1.000 patients with chronic conditions will be screened over 24 months with questionnaires for anxiety (GAD-7), depression (PHQ-9), and adherence problems (MARS-D). We expect to find at least N=162 adolescents with suspicious screening results participating in the study, which will be advised to seek psychological care. Either a physician trained in MI, who will offer an additional second appointment to talk about the screening results, or an untrained physician who performs treatment as usual (TAU) within the clinical visit will give recommendations on the day of the screening.
MI or TAU will be both performed by about 15-17 randomized treating physicians of the University children’s hospital in each group.

Study details:
Medical research in connection with medical care

COACH Consortium:
The study is part of a BMBF funded (Federal Ministry of Education and Research, Germany) healthcare project, the COACH consortium: Chronic Conditions in Adolescents: Implementation and Evaluation of Patient-centered Collaborative Healthcare.
Further information will be available online: https://www.coach.klips-ulm.de/de/was-ist-coach/

Study design:
Pragmatic cluster-randomized, monocentric controlled trial, two parallel groups; Physicians of the patients will be randomized

Study site:
Monocentric study at the
University Children´s Hospital Düsseldorf
Department of General Pediatrics, Neonatology and Pediatric Cardiology
Heinrich-Heine-University Düsseldorf
Moorenstr. 5, 40225 Düsseldorf, Germany

Study team:
Principal investigator (PI):
Prof. Dr. med. Thomas Meissner, MD; thomas.meissner@med.uni-duesseldorf.de
University Children’s Hospital Düsseldorf
Department of General Pediatrics, Neonatology and Pediatric Cardiology
Heinrich-Heine- University Düsseldorf.
Moorenstr. 5, 40225 Düsseldorf, Germany

Study mobile phone: available after study initiation
Study E-mail: coach-mi@med.uni-duesseldorf.de

Study physicians:
Katharina Förtsch; katharina.foertsch@med.uni-duesseldorf.de
Dr. med. Hannah Linderskamp, MD; hannah.linderskamp@med.uni-duesseldorf.de
Dr. med. Christina Reinauer, MD; christina.reinauer@med.uni-duesseldorf.de

Research assistants:
Yasemin Fidan, study nurse; yasemin.fidan@med.uni-duesseldorf.de
Rabea Viermann, research assistant; rabea.viermann@uni-duesseldorf.de
Marina González Biber, research assistant
Anna Lena Platzbecker, research assistant

Objectives:

1. The primary outcome is the utilization of mental healthcare within six months post-intervention.
2. Multiple secondary outcomes such as the number of sessions attended, disease-specific illness parameters, adherence problems, missed clinical visits, symptoms of anxiety and depression will be assessed.
3. Examining possible serious adverse events (SAEs) associated with the present screening/MI approach.

The overall goal of our project is to improve entry into mental healthcare by psychiatrists, psychologists, or psychotherapists for those adolescents in need of support. The long-term implementation of MI for adolescents with different chronic diseases might serve as a model to optimize healthcare management in daily clinical routine.

Data collection:

First contact:
Patients who have a scheduled appointment in the specialized outpatient clinic are informed by telephone contact about the study several days before the appointment. If they are interested to participate in the study, study information and the informed consent form are sent to the family by e-mail or regular mail. Informed consent can be given for this study at our institution and separately, if eligible, for other projects within the COACH consortium (separate informed consent forms). There will be the option for a further agreement on voice recording of the MI or TAU interview for a qualitative analysis of the conversation.

Patient visit:
The next contact will be at the outpatient clinic of the University Children's hospital Düsseldorf right before the clinical appointment with the treating physician of the outpatient clinic. Patients and parents will be invited to join in the COACH-MI study and to discuss the study with study physicians. All patients will be asked to answer the newly established screening questionnaires for anxiety, depression, and adherence on a tablet computer. The screening will be performed once every year in all patients with chronic conditions as part of a newly implemented internal standard procedure of our institution, and participation in the study will be optional.

If patients and parents decide to participate in the study and the informed consent form is signed, the screening can start if:
a) Both parents and the patient signed the informed consent form and security of data agreement
b) The patient is ≥18 years old and has signed the informed consent form and security of data agreement
c) The patient and one parent signed, and the signing parent agreed to get the consent of the second parent. The signature of one parent is sufficient to start the study if he/she asserts that participation in the study is the supposed wish of the second parent. If the second parent disagrees participation will be terminated and all data will be deleted.
d) The adolescent patient (12-18 years) regularly visits the outpatient clinic alone and he signs the informed consent form. In this case, the written parental agreement has to be gained within the following two weeks. Without the parental agreement, the patient has to be excluded and his data have to be deleted. The signature of one parent is sufficient if he/she asserts that participation in the study will be the wish of the second parent. If the second parent disagrees, participation will be terminated and all data will be deleted.
The usual process will be to gain informed consent at least 24 hours before the clinical visit. Only, if this was not possible, informed consent on the day of the clinical visit will be accepted as an alternative approach.

After completing the screening questionnaires, the Patient Health Questionnaire (PHQ-9), the 7-item Generalized Anxiety Disorder scale (GAD-7), and the adherence questionnaire (MARS-D), using a secured program on a tablet PC, immediate feedback on the results is provided to the treating physicians of the outpatient clinic (after login with a keyword). The result will be demonstrated and explained to the patient and caregivers by the treating pediatrician.
Results of GAD-7 and PHQ-9 will be defined as screening positive if the outcome is ≥7 points in one or both of the questionnaires. Alternatively, a positive item 9 in PHQ-9 (self-harm or suicidal ideations, score >=1) defines a positive screening result. This will trigger the recommendation to seek mental healthcare. As there is no established threshold for non-adherence in the MARS-D questionnaire (Tommelein et al., 2014), the results of the MARS questionnaire will not trigger a positive screening result.
In cases where screening in the abovementioned questionnaires is inconspicuous but the treating physician sees a need for psychologist counseling for reasons other than anxiety, depression, or non-adherence, the treating physician can individually decide whether he recommends psychologist counseling. Physicians will be asked to document reasons for their recommendations on a report form.

MI and TAU are both performed by the patients' treating physicians. The treating physicians in the different outpatient clinics of our institution are randomized to either MI or TAU before the study recruitment. In every outpatient clinic, there will be several physicians that either do MI or TAU.
MI physicians will be asked for confidentiality about MI (written informed consent) and their new way of facilitating patients´ motivation to prevent contamination bias. Both groups of treating physicians will be asked to document details on their conversations and monitor whether clinical management changed over time.

The cluster-randomization of the physicians will be performed after the informed consent of the participating physicians at the Institute of Epidemiology and Medical Biometry at the University of Ulm (p 6, 10). Physicians randomized to MI will complete a certified, two-day education course in MI prior to the study start. They will use the MI technique in the first appointment on the screening day. MI physicians will offer/recommend a second appointment within the following two weeks to talk again about motivation, the test results, and a potential need for psychological support (see below).
All patients with conspicuous screening results (both MI and TAU) will receive standardized written feedback on the screening visit day and direct brief advice to seek further supportive psychological counseling. This will include contact addresses of the local psychological appointment systems ("Termin Service Stelle" and "Zentrale Informationsbörse Psychotherapie (ZIP)" of the Kassenärztliche Vereinigung Nordrhein) to arrange an appointment with a psychotherapist treating adolescents within the area of Düsseldorf and internet address of a Search Engine for psychotherapist treating adolescents within the area of Düsseldorf. Moreover, it will include the telephone number of the local psychological-social care service in the SPZ "Sozialpädiatrisches Zentrum" at the University Children's Hospital.
Patients screened positive for anxiety or depression will have the option to contact the colleagues of another COACH subproject and to agree to participate in a study on resources and adaption (further questionnaires) or internet- and mobile-based cognitive behavioral therapy (iCBT, both needing separate informed consent), provided that the other COACH studies are ongoing and recruiting. A separate ethics vote will be obtained by the University of Ulm and Potsdam investigators and will be submitted to our Ethics Committee for agreement before this option is offered to patients.

Disease-specific illness parameters indicating disease severity, such as HbA1c, lung function testing (FEV expected), body mass index, and other-disease related parameters (e.g., needing a wheelchair, needing oxygen) will be obtained from the medical record and included in the Case Report Form (CRF). The TAU and MI sessions (screening day and following MI visit) can be voice recorded after an optional, separate agreement of participation of patients, parents, and physicians. All physicians performing MI will complete a short questionnaire on the outcome of the interview and use of MI after each patient interview (questions will become available after MI Training, see *Addendum*, p11). We aim to ensure the correct use of MI and to evaluate whether physicians use this technique in their conversations.

Follow-up (six months after MI/TAU):
Six months after inclusion in the study, participants will be contacted by telephone interview and/or by e-mail to participate in the follow-up re-evaluation. Patient-reported outcomes will be obtained by telephone interview and via online questionnaires, which the patient will receive as a link by e-mail. The follow-up telephone interview (or face to-face-interview as part of a regular outpatient appointment) will be performed as a semi-structured interview on healthcare utilization. This interview will gather detailed information on whether patients tried to make an appointment with a psychologist/psychotherapist/other mental health services, whether they did have appointments and how many, or what were the reasons to not seek counseling and thereby enable us to evaluate which mental health support was used.
Investigators will also check for AEs and SAEs that were not previously reported to the study team.

An annual mental health screening on anxiety and depression will be implemented as a clinical measure for all adolescents with chronic conditions, thus, all patients will be asked to join the rescreening 12 months after inclusion and will be asked for their agreement to evaluate these follow-up data as part of the study. Patients will be invited to again complete the initial mental health screening questionnaires. Questionnaires may be completed either in the outpatient department on a tablet computer (as part of a regular outpatient appointment), on paper pencil, on a tablet computer, or via online questionnaires.

Sample Size estimation:

The proposed sample size for the mental health screening is estimated to be about N=1.000 in the 24 months recruitment period. The prevalence of symptoms of depression and anxiety is estimated at 20-25% in total (Quittner, Saez-Flores, & Barton, 2016). We, therefore, expect to find at least N=200 patients with conspicuous screening results. Exclusion criteria are depicted below, or some patients may decide not to participate.

The rate of successful referrals to mental healthcare in usual care is estimated to be 10%, and we expect an increase of up to 30% in the intervention group. A Chi-square test will be applied to compare both groups. The sample size software NQuery Advanced 8.1 (Statistical Solutions, Ireland) for the two-sided chi-square test, power 80% and a significance level of 5% gives a sample size of N=62 in each group. Correcting for the cluster structure of the trial we assume a mean cluster size of 5 patients per physician. An ICC is not known. We will correct the sample size by 10% for cluster effects resulting in an estimated ICC of 2.5% and a sample size of N=69 per group. To adjust for 15%, drop out (see next paragraph), a sample size for each group is estimated as N=81 treated by MI physicians or TAU physicians, summing to a total of 162 patients. A mean cluster size of 5 patients per physician results in a sample size of physicians as n=34 (2 x 17).

Compliance / Rate of loss to follow-up:

About 15% were lost to follow-up after the initial screening in a comparable study (Dean, Britt, Bell, Stanley, & Collings, 2016). Follow-up rates are reported in the range between 100% and 68% in the study of Van Voorhees et al. (2009) and 70% for follow-up data after one year (Saulsberry et al., 2013). All cases once randomized to the study will be analyzed in an intention-to-treat analysis (ITT, Figure 1).

To be assessed for eligibility:

Screening for depression, anxiety, or non-adherence:
Aim: N>1.000
To be allocated to trial: N=162
To be analyzed: N=162

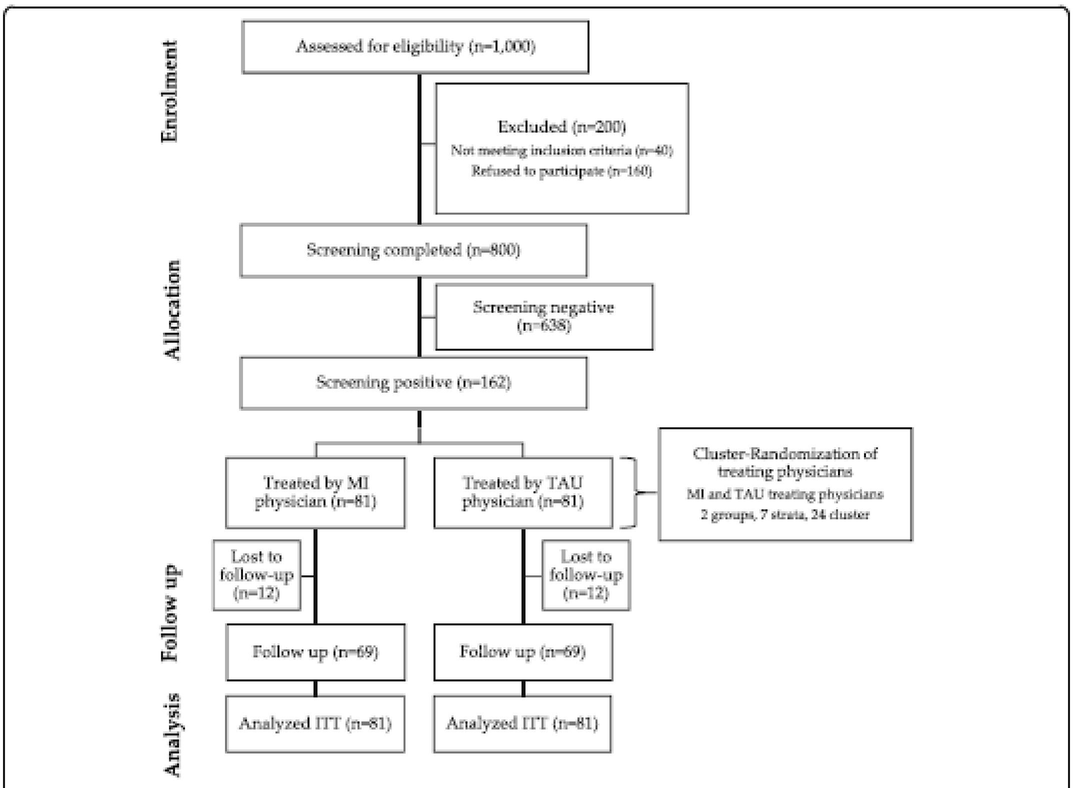

Fig. 1 Flow chart of study design. ITT intention to treat, MI motivational interviewing, TAU treatment as usual

Figure 1: Flow chart of study design. ITT intention to treat, MI motivational interviewing, TAU treatment as usual (from Reinauer et al., 2018)

<u>Data / Statistical Analysis:</u>

Primary outcome (Follow-up):

The primary outcome is the utilization of psychological healthcare, e.g., psychotherapy, psychological counseling, or iCBT, defined as making at least one appointment face-to-face or digitally within 6 months after inclusion in the study (dichotomous variable). Those patients who are on a waiting list are recorded separately. The primary outcome will be assessed via a semi-structured interview on healthcare utilization (follow-up).

Secondary outcomes:

The secondary outcomes are:

- Number of face-to-face sessions (or online intervention) with a psychologist/psychological psychotherapist/iCBT attended within the 6 month follow-up interval (as recorded in the interview)
- Changes in disease-specific illness parameters, such as HbA1c, lung function testing, body mass index, if available
- Missed clinical visits, as an additional behavioral measure for adherence (assessed by records of the hospital)
- Symptoms of anxiety (GAD-7)
- Symptoms of depression (PHQ-9)
- Self-reported adherence problems (MARS-D)
- Follow-up interview on healthcare utilization and the reasons for not claiming psychological counseling after MI or TAU
- Quantitative and qualitative analysis of MI/TAU conversations.

Statistical Analysis:

The analysis of the main outcome will use a logistic mixed model adjusting for the cluster structure in the data, adjusted for confounding factors, such as age and gender. The analyses will be done in the ITT population.

The secondary measures will be analyzed with:

- Non-parametric test

- The number of psychological face-to-face sessions or online intervention sessions attended within the 6-month follow-up interval – without 0 sessions because then the primary outcome will influence this secondary outcome.
  - Missed clinical visits as a measure for adherence.
  - Acceptance to participate in the study (gender comparison).
- Mixed ANOVAs
  - Symptoms of anxiety, depression, non-adherence (GAD-7, PHQ-9, MARS-D).
  - Specific illness parameters, such as HbA1c, lung function parameters, body mass index, etc., if available.
  - The safety of the treatment will be analyzed by comparing the rates of SAEs/AEs between groups by Chi-square-test or exact Fisher test (depending on the frequency).

<u>Intervention Scheme:</u>

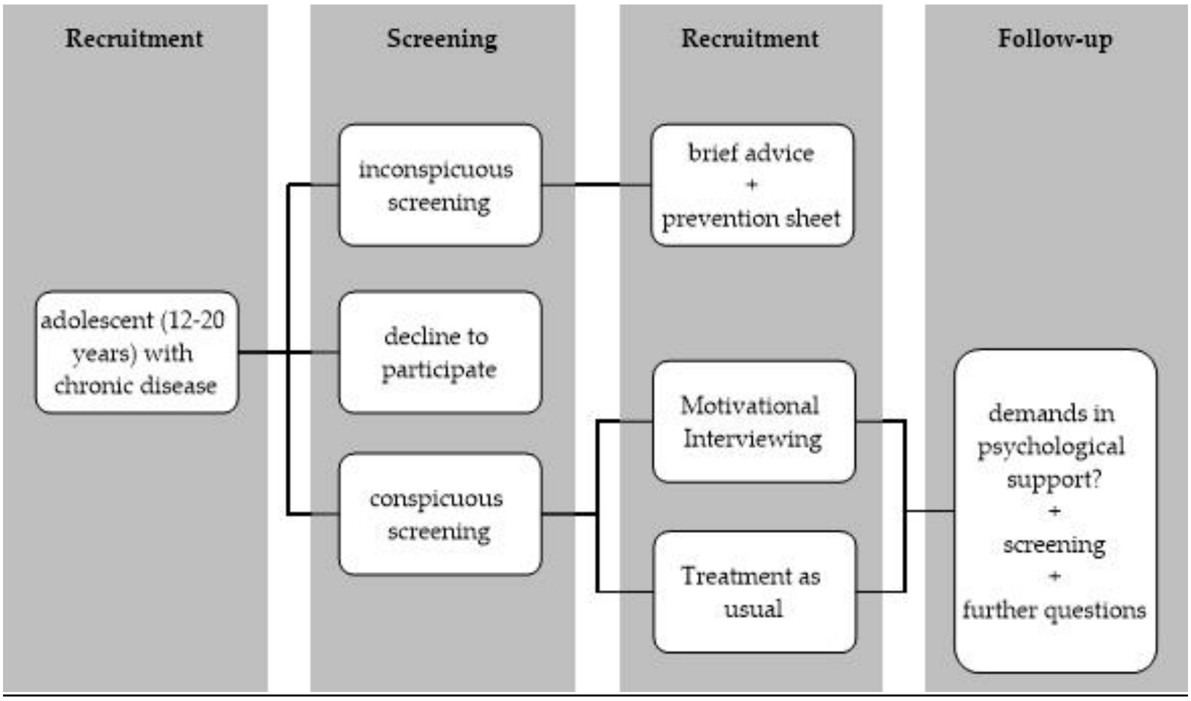

Figure 2: Intervention Scheme

In the recruitment phase, screening data of adolescents with chronic conditions will be obtained with screening questionnaires at the specialized outpatient departments of the University Children's Hospital Düsseldorf. After providing their informed consent, patients complete the screening questionnaire on a tablet computer (Figure 2) The study makes use of the screening tool, which is programmed to give an immediate evaluation (after login of the treating physician). Screening includes the Generalized Anxiety Disorder 7-item scale (GAD-7; Spitzer, Kroenke, Williams, and Löwe (2006)), the depression part of Patient Health Questionnaire (PHQ-9; Kroenke, Spitzer, and Williams (2001)) and questions on adherence to therapy (MARS-D). Either a GAD-7 or PHQ-9 score ≥7 will be defined as an indication of the need for supportive counseling.

Those patients with inconspicuous screening results will be reassured that there is good adaption and get brief advice that there is currently no need for psychological care. They will receive Lowe a psychoeducational handout about helpful resources and about coping with chronic conditions.

Patients with positive screening results will constitute the intervention group. Their treating physicians were previously randomized to MI or TAU. Depending on the physician's training status, patients are allocated to the MI or TAU group. Patients with conspicuous screening results will be advised by an MI-trained physician or a TAU physician (depending on his/her randomization) to consult a psychologist or psychotherapist.
Physicians advise screening-positive patients to seek psychological support: Patients with both MI or TAU get a written recommendation - an information sheet about therapeutic options: Psychotherapists at the on-site social pediatric center (SPZ) or seeing a psychotherapist practitioner, psychiatrist, or clinical psychologist.

“MI patients” receive an immediate extensive talk and advice (MI technique), and a second MI session within two weeks is recommended. This optinal, offered second appointment should be regularly scheduled to be a second face-to-face visit in the outpatient clinic, and only if not accepted by the patient in exceptional cases an alternative telephone session with the treating physician will be offered.
The control condition is immediate brief advice which represents TAU on the screening day visit. The topic and length of this advice will be not defined or structured and should represent the usual care of the attending physician. To describe TAU, usual care visits will be documented by physicians (length, topics) and can be audio recorded to examine the extent of natural/spontaneous MI communication strategies used in the control condition.

Six months after study inclusion the patients will be interviewed about their mental healthcare utilization at follow-up. The primary outcome is the successful uptake of a mental healthcare intervention within the 6-month follow-up interval. A reassessment of secondary outcomes will be performed at follow-up and annual rescreening. Longitudinal clinical data will be obtained at the most recent regular visit.

Intervention training: Education of physicians in MI

The intervention is to establish a Motivational interviewing (MI) training to enable physicians to deliver an MI-based ultra-short intervention aiming at patients´ motivation towards claiming mental healthcare by psychiatrists, clinical psychologists, or psychotherapists.
MI and TAU are both performed by the treating physician of the patients in the outpatient clinic setting. There are different outpatient clinics at our institution involved.
Physicians will be cluster-randomized to either MI or TAU before the study, using the Minimization procedure of Pocock and Simon (Pocock and Simon, 1975) using the randomization software RITA, Version 1.31 (RITA (Randomization In Treatment Arms), 2013). As prognostic factors/strata, the medical specialization of the physicians and the expected cluster size (small, big) will be considered. A mean number of about 5 patients per physician is assumed. The randomization will be performed with the informed consent of the participating physicians in an independent institution (Institute of Epidemiology and Medical Biometry at the University of Ulm).
Physicians will be trained in MI in a two-day workshop by an experienced, certified psychologist from the “Motivational Interviewing Network of Trainer” (MINT) organization (GK Quest Akademie, Heidelberg). The training consists of basic information about MI, practical aspects, practical training, and an elaboration of a checklist for the following MI in the study. This checklist, Motivational Interviewing Treatment Integrity (MITI) can be implemented to ensure that the physicians make use of important aspects of MI during the interview.
The practical training includes videotaping of a “training proband” following discussion with an expert. Additional telephone supervision of MI can be utilized with an expert from GK Quest Akademie GmbH. A study from Miller, Yahne, Moyers, Martinez, and Pirritano (2004) demonstrated, that clinicians can be successfully trained in a two-day course in MI. Refreshment group training will be organized within the study period of 24 months.

Addendum:

The two-day MI workshop was conducted with a focus on chronic conditions, anxiety, and depression symptoms and on how to improve the use of mental healthcare services. Topics included theoretical definition and core principles of MI and skill practice, focusing on stages of change, empathy, MI spirit, exploration of ambivalence, rolling with resistance, change talk, and confidence talk. Participants worked on a brief guide for their MI consultations after anxiety and depression screening. Participants were provided a pocket guide on MI-consistent conversation techniques, e.g., open-ended questions, reflective listening, affirmations, advice with permission, creating collaboration, and emphasizing autonomy/control. , a flowchart for the conversation structure and helpful phrases in the context of CMCs. The course contained supervised, practical exercises for a focused MI intervention, aiming to improve access to mental healthcare in adolescents.

To achieve a high external validity, we will try to include all patients from the outpatient department of the University children’s hospital fulfilling the inclusion criteria and randomize all the physicians who take care of these patients after they gave informed consent to randomization. We aim at improving the acceptance of psychological support. We consider it most reasonable to teach the treating physicians who regularly see the patients with chronic conditions in the outpatient clinic. They are the familiar healthcare professionals and thus the gatekeepers towards mental healthcare offers. An alternative model including a psychologist or external counselor for the delivery of MI would not represent usual clinical practice and limits acceptance, future dissemination, and implementation. By training the treating physicians in MI we also expect to establish sustainable effects towards better skills of physicians in patient-centered communication with their adolescent patients.

Controls:

We decided to use TAU as the comparator. Physicians randomized to TAU are not trained in MI and are asked to give standard advice on the screening results, including the recommendation to consult psychological care. TAU will be captured by a standardized physician report to allow for a description of TAU (length, content, topics, etc.).

Trial Duration:

Time for preparation of the trial (months): 6 months
Recruitment period (months): 24 months
First subject / patient in to last subject / patient out (months): 36 months
Time for data clearance and analysis (months): 9 months
Duration of the entire trial (months): 48 months
Expected start of recruitment: April 2018

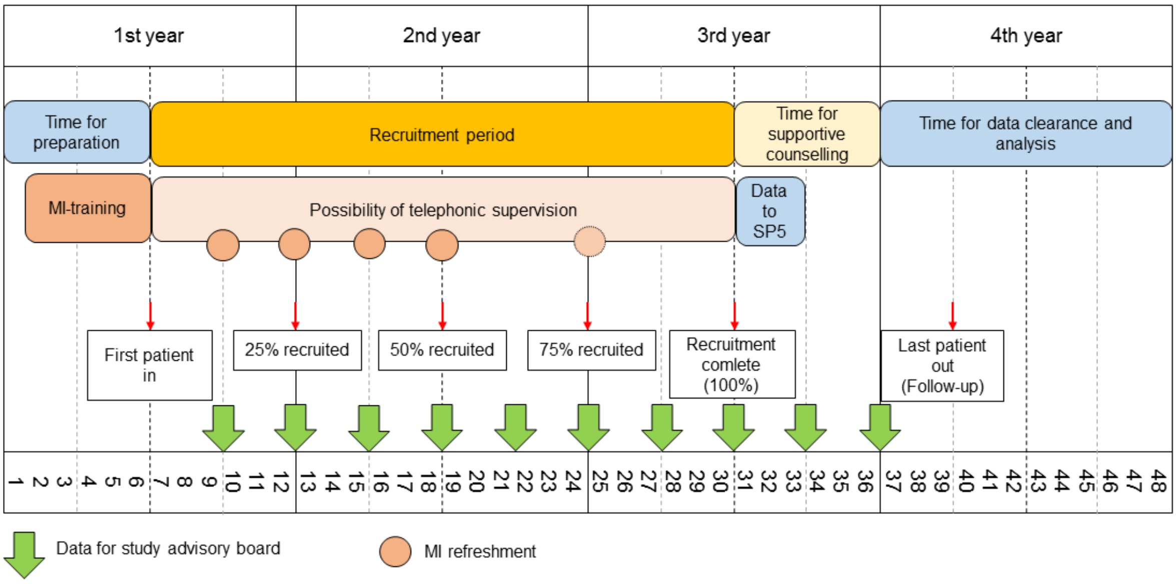

Figure 2: *Trial flow*

Target Population and Inclusion / Exclusion Criteria:

Inclusion criteria: Age 12-20 years; chronic medical condition. Chronic medical conditions are defined as diseases that have one or more of the following characteristics: duration of condition > 1 year, causing significant impairment of daily routine, need for continuous care, and/or medical treatment.
This will include the following diseases: Asthma, allergy, cystic fibrosis, diabetes, endocrine disorders (adrenal insufficiency, growth hormone deficiency, thyroid disorders, metabolic syndrome), neurological diseases (epilepsy, multiple sclerosis, migraine, cerebral palsy, condition after stroke, brain tumors, hydrocephalus), chromosomal disorders (trisomy), heart disease (congenital heart disease, cardiomyopathy, arrhythmia, hypertension), inborn errors of metabolism (phenylketonuria, glycogen storage disease, organic acid disorders, galactosemia, severe hyperlipidemia, congenital hyperinsulinism), chronic inflammatory bowel disease, celiac disease, chronic abdominal pain, hepatopathy, short bowel syndrome, arthritis, lupus, autoinflammatory diseases, HIV, Aids.
Exclusion criteria: Current psychotherapy, psychosis, acute suicidality, severe cognitive deficit, communication/language problems.

Ethical Considerations/Good Clinical Practice:

The study will be performed following the principles of Good Clinical Practice (GCP), with the Helsinki protocol, and with all current ethical standards. The study protocol will be approved by the Ethics Committee of the University of Düsseldorf (COACH-MI study). This will include the formal informed consent procedures with the caregivers/legal guardians of adolescent patients, and informed consent from the patients 18-20 years old as described above. The participants will be informed that they can leave the respective study at any time without any disadvantages for their medical care.

Research participants will get easier access to mental healthcare screening and discussion of this screening with the physician compared to usual care. Results of screening measures will be reported to the patients and their caregivers. The intervention (education of the treating physician in MI to improve entry into mental healthcare use) is considered safe.

A standard procedure to respond to suicidal ideations or suicidal behaviors of study participants will be integrated into the teaching of study physicians and treating physicians before the study start. If physicians suspect suicidality during the study, they are advised to use the Columbia Suicide Severity Rating Scale (C-SSRS, http://www.cssrs.columbia.edu) as a standard tool for the evaluation of suicidality. Alternatively, they can immediately include the in-house psychological team on the same day for evaluation of suicidality. If there is substantiated concern of suicidality, the patient will be transferred to the local psychiatry center for evaluation.

Data safety will be assured following the German data protection regulations.

Since the intervention of this study is one or two low-intensity MI sessions provided by MI-trained physicians, we do not expect any SAEs concerning the intervention condition. However, the implementation of a yearly screening for anxiety and depression and the evaluation of adherence problems, and a resulting recommendation to seek psychological care might have negative effects on patient well-being. Potential specific AEs/SAEs are related to unmasking dormant conflicts: worsening of anxiety, depression, and/or adherence with the resulting need of admission to the hospital. Therefore, we will record and analyze the occurrence of AEs/SAEs next to the data mentioned during the study and at follow-up (six months after MI/TAU).
As soon as SAEs get to the knowledge of the investigators, they will be reported within 24 hours to the PI in Düsseldorf (Prof. Meissner or substitute) and the study team of the University of Ulm (Prof. Baumeister or substitute) who will assess the relation to study intervention and directly inform the DSMB (related) or include the SAE in the regular reports to the DSMB (unrelated).

Benefit-risk balance:
A mental health screening of adolescents with chronic disease is not regularly implemented today. Such screening is regarded as an important improvement of current clinical practice to help identify those patients with a need for additional psychological support in the difficult phase of adolescence. Anxiety and depression may lead to non-adherence, harmful behavior, and insufficient treatment in the affected adolescents.
Education of physicians in MI has also to be regarded as an improvement of physicians' skills for the benefit of the patients. Both measures are considered safe without major risk for study participants. Even if screening may trigger a temporary deterioration by unmasking dormant conflicts, we do believe that detecting depression and anxiety in adolescents with chronic conditions is an important measure to ensure good care and treatment.
The most likely safety concern for the patients might be that former unknown suicidality or severe depression is uncovered by the screening testing or the following advice (TAU/MI).

Insurance:
In cases where a second appointment is scheduled for MI, travel accident insurance for the participants and their caregivers for going to and from the site is provided (Marsh Medical consulting).

Quality Assurance, Safety, Data Protection and maintenance of Medical Confidentiality
Mental health screenings will be generally implemented with an internal Standard Operating Procedure (SOP) at the Children's Hospital, guiding clinicians on how to screen adolescents with chronic disease. Recruitment of suitable patients will be carried out with the assistance of the study team. Established and validated questionnaires will be used.
MI education of the physicians by certified trainers will be performed with a specialized institute (GK Quest Akademie) with experience in teaching MI (MI Network of Trainers, MINT). MINT is an international organization, with the overall aim to ensure and improve the implementation and quality of MI. Evaluation of MI competence can be analyzed with an established coding system for MI (MITI 4.2; German version, Motivational Interviewing Treatment Integrity check). Furthermore, during the study period, additional telephone counseling and supervision are offered. After the basic training regular "booster-teaching" will be offered.

Addendum:
Conversation length and semiquantitative use of MI-consistent techniques are self-reported by the physicians after each consultation in a short written questionnaire. MI physicians report their semi-quantitiave use of six basic MI techniques: advice with permission, open-ended questions, reflective listening, affirmations, creating collaboration, and emphasizing autonomy/control; from 0=not used to 2=often used, for a maximum score of 12 points.

The Department of Clinical Psychology and Psychotherapy, Institute of Psychology and Education, University of Ulm, and the Institute of Epidemiology and Medical Biometry at the University of Ulm will provide methodical trial support,

including independent randomization of participating physicians, data management, collecting reports for the DSMB and statistical expertise in the evaluation of study data as follows:

1. All data of the electronic screening process (pseudonymized questionnaires) will be stored on the local server of the University of Düsseldorf or if this will not be possible on a secured German server, fulfilling the current data safety regulations in Germany. Patient-related data are deposited on a protected local database on the server of the University of Düsseldorf in pseudonymized form. Informed consent forms (written, signed, and dated) and case report forms are locally stored in hardcopy in the investigator site file (ISF) for each patient with limited access to ensure confidentiality and security. Access to the study files will be limited to the study physicians. Anonymized data reports may be sent to the University of Ulm for statistical evaluation, using appropriate data protection measures which will be consented to with data protection experts from the COACH consortium at the University of Ulm (Prof. H. Baumeister, Department of Clinical Psychology and Psychotherapy, Institute of Psychology and Education, University of Ulm and Prof. R. Holl, Institute of Epidemiology and Medical Biometry at the University of Ulm). An ethics vote of the University of Ulm will be obtained before study data transfer and sent to the Research Ethics Committee of the University of Düsseldorf for evaluation.
2. Data of primary and secondary outcome parameters will be obtained during initial and following clinical visits or by follow-up telephone interview and will be stored in pseudonymized form locally in Düsseldorf and transferred to the coordinating study center at the University of Ulm as described above.
3. An independent Data Safety and Monitoring Board will support the investigators during the study. The coordinating center will collect and forward reports for the intervention studies of the consortium for the scheduled scientific advisory board meetings.
4. For GCP conformity SAEs will be reported within 24 hours to the PI in Düsseldorf (Prof. Dr. Meissner or substitute) and the central coordinating study team of the University of Ulm (Prof. Dr. Baumeister or substitute). Both institutions will assess whether the SAE is "clearly related" to the intervention (MI), "maybe related" or "unrelated". The DSMB will be immediately informed about related/maybe related SAEs and decide on continuation, modification, or stop of study.

Data Safety and Monitoring Board (DSMB):

A Data Safety and Monitoring Board (DSMB) for the COACH consortium with independent experts from Pediatrics and Psychology will oversee the study. Quarterly progress will be reported to the DSMB. DSMB members will evaluate the progress and safety of the study and decide about continuation or modification.

Questionnaires:

Generalized Anxiety Disorder Screener (GAD-7): The Generalized Anxiety Disorder Screener is a practical self-report anxiety questionnaire that consists of 7 items and describes the most prominent diagnostic features of the DSM-V diagnostic criteria A, B, and C for generalized anxiety disorder. The 7 core symptoms of generalized anxiety disorder can be scored from 0 = "not at all" to 3 = "more than half the days" during the last two weeks. The GAD-7 scores range from 0 to 21 and the cut-off points of 5, 10, and 15 represent the thresholds for mild, moderate, and severe anxiety symptom levels, respectively (Löwe et al., 2008). For the GAD-7, good internal consistency is reported with Cronbach's α .79 and .91 (Dear et al., 2011). For the current study, a cut-off of ≥7 will be used as a qualifier for inclusion in the study.

Patient Health Questionnaire (PHQ-9): The PHQ-9 (Kroenke & Spitzer, 2002) is a 9-item depression module from the full Patient Health Questionnaire that can be entirely self-administered by the patient. In the PHQ-9, each of the 9 DSM-V criteria can be scored from 0 = "not at all" to 3 = "nearly every day". The range of the sum-cores is from 0 to 27 and the cut-off points of 5, 10, 15, and 20 represent the thresholds for mild, moderate, moderately severe, and severe depression, respectively (Kroenke & Spitzer, 2002; Kroenke et al., 2001). For the PHQ-9, internal reliability estimates range from .86 to .89 using Cronbach's α. Two-day test-retest reliability is estimated to be .84 with nearly identical mean total scores (Kroenke et al., 2001). For the current study, a cut-off of ≥7 will be used as a qualifier for inclusion in the MI study.

Medication Adherence Rating Scale (MARS-D): The Medication Adherence Rating Scale is a five items questionnaire assessing adherence to medical treatment (Mahler et al., 2010; Thompson et al., 2000). Five questions ask about both intentional and unintentional nonadherence, items are Likert scale ranging from 1 to 5 resulting in a total score between 5 and 25, (Mahler et al., 2010; Thompson et al., 2000), 25 points resemble adherence, if patients score lower, the pathological items will be displayed to the treating physician to consider together with the patients whether non-adherence jeopardizes health.

References:

Dean, S., Britt, E., Bell, E., Stanley, J., & Collings, S. (2016). Motivational interviewing to enhance adolescent mental health treatment engagement: a randomized clinical trial. Psychological Medicine, 46(09), 1961-1969.

Dear, B. F., Titov, N., Sunderland, M., McMillan, D., Anderson, T., Lorian, C., & Robinson, E. (2011). Psychometric comparison of the generalized anxiety disorder scale-7 and the Penn State Worry Questionnaire for measuring response during treatment of generalised anxiety disorder. Cognitive behaviour therapy, 40(3), 216-227.

Horne R., Weinman J. Self-regulation and self-management in asthma: exploring the role of illness perceptions and treatment beliefs inexplaining non-adherence to preventer medication. Psychol Health.2002;17(1):17–32.24.

Kroenke, K., & Spitzer, R. L. (2002). The PHQ-9: a new depression diagnostic and severity measure. Psychiatric annals, 32(9), 509-515.

Kroenke, K., Spitzer, R. L., & Williams, J. B. (2001). The Phq-9. Journal of general internal medicine, 16(9), 606-613.

Löwe, B., Decker, O., Müller, S., Brähler, E., Schellberg, D., Herzog, W., & Herzberg, P. Y. (2008). Validation and standardization of the Generalized Anxiety Disorder Screener (GAD-7) in the general population. Medical care, 46(3), 266-274.

Mahler, C., Hermann, K., Horne, R., Ludt, S., Haefeli, W. E., Szecsenyi, J., & Jank, S. (2010). Assessing reported adherence to pharmacological treatment recommendations. Translation and evaluation of the Medication Adherence Report Scale (MARS) in Germany. Journal of evaluation in clinical practice, 16(3), 574-579.

Miller, W., Yahne, C., Moyers, T., Martinez, J., & Pirritano, M. (2004). A Randomized Trial of Methods to Help Clinicians Learn Motivational Interviewing. Journal of consulting and clinical psychology, 72(6), 1050-1062.

Pocock, S. J., Simon, R. (1975): Sequential treatment assignment with balancing for prognostic factors in the controlled clinical trial, Biometrics 31, 103—115

Quittner, A. L., Saez-Flores, E., & Barton, J. D. (2016). The psychological burden of cystic fibrosis. Current opinion in pulmonary medicine, 22(2), 187-191.

Reinauer, C., Viermann, R,, Förtsch K., Linderskamp H., Warschburger P., Holl R.W., Staab D., Minden K., Muche R., Domhardt M., Baumeister H., Meissner T. (2018). COACH consortium. Motivational Interviewing as a tool to enhance access to mental health treatment in adolescents with chronic medical conditions and need for psychological support (COACH-MI): study protocol for a cluster randomised controlled trial. Trials, 14;19(1):629.

RITA (Randomization In Treatment Arms) Software, Vers. 1.31 (2013) Evidat - Statistical Apps + Consulting, Dr. Friedrich Pahlke / Lübeck

Saulsberry, A., Marko-Holguin, M., Blomeke, K., Hinkle, C., Fogel, J., Gladstone, T., Van Voorhees, B. W. (2013). Randomized Clinical Trial of a Primary Care Internet-based Intervention to Prevent Adolescent Depression: One-year Outcomes. Journal of the Canadian Academy of Child and Adolescent Psychiatry, 22(2), 106-117.

Spitzer, R. L., Kroenke, K., Williams, J. B., & Löwe, B. (2006). A brief measure for assessing generalized anxiety disorder: the GAD-7. Archives of internal medicine, 166(10), 1092-1097.

Statistical Solutions. (2015). nQuery Advisor + nTerim 4.0 Users Guide. Cork, Ireland. Retrieved from http://www.statsols.com/nquery-sample-size-calculator

Thompson, K., Kulkarni, J., & Sergejew, A. (2000). Reliability and validity of a new Medication Adherence Rating Scale (MARS) for the psychoses. Schizophrenia research, 42(3), 241-247.

Tommelein E, Mehuys E, Van Tongelen I, Brusselle G, Boussery K. Accuracy of the Medication Adherence Report Scale (MARS-5) as a quantitative measure of adherence to inhalation medication in patients with COPD. Ann Pharmacother. 2014 May;48(5):589-95.

Van Voorhees, B. W., Fogel, J., Pomper, B. E., Marko, M., Reid, N., Watson, N., Domanico, R. (2009). Adolescent Dose and Ratings of an Internet-Based Depression Prevention Program: A Randomized Trial of Primary Care Physician Brief Advice versus a Motivational Interview. Journal of cognitive and behavioral psychotherapies: the official journal of the International Institute for the Advanced Studies of Psychotherapy and Applied Mental Health, 9(1), 1-19.
