# Supplementary material for: Efficacy of Motivational Interviewing to Improve Utilization of Mental Health Services Among Youths With Chronic Medical Conditions: A Cluster Randomized Clinical Trial
Source: JAMA Netw Open. 2021 Oct 1;4(10):e2127622. doi: 10.1001/jamanetworkopen.2021.27622 (PMC8486984; doi:10.1001/jamanetworkopen.2021.27622)
Supplement: Supplement 2. — Data Sharing Statement [file jamanetwopen-e2127622-s002.pdf]

Data Sharing Statement

Reinauer C, Platzbecker AL, Viermann R, et al. Efficacy of Motivational Interviewing to Improve Utilization of Mental Health Services Among Youths With Chronic Medical Conditions. JAMA Netw Open. 2021;4(10):e2127622. doi:10.1001/jamanetworkopen.2021.27622

Data
Data available: No

Additional Information
Explanation for why data not available: Data generated during this study are not publicly available because the participants did not agree for their data to be shared publicly. Individual de-identified, anonymized data are available from the authors upon reasonable request (https://www.coach.klips-ulm.de/).
